# *MYCN* is a novel oncogenic target in adult B‐ALL that activates the Wnt/β‐catenin pathway by suppressing *DKK3*

**DOI:** 10.1111/jcmm.13644

**Published:** 2018-04-19

**Authors:** Desheng Kong, Linlin Zhao, Lili Sun, Shengjin Fan, Huibo Li, Yanqiu Zhao, Zhibo Guo, Leilei Lin, Lin Cui, Ke Wang, Wenjia Chen, Yihui Zhang, Jin Zhou, Yinghua Li

**Affiliations:** ^1^ Department of Hematology The First Affiliated Hospital Harbin Medical University Harbin China; ^2^ Department of Hematology The Fourth Affiliated Hospital Harbin Medical University Harbin China; ^3^ Department of Blood Transfusion The First Affiliated Hospital Harbin Medical University Harbin China

**Keywords:** 5‐AdC, adult B‐cell acute lymphoblastic leukaemia, DKK3, MYCN, Wnt/β‐catenin

## Abstract

*Dickkopf‐3* (*DKK3*) is frequently down‐regulated by promoter hypermethylation and is closely associated with a poor prognosis in many cancers. Our previous studies have shown that *miR‐708* down‐regulates *DKK3* at the post‐transcriptional level in B‐ALL. However, whether transcriptional mechanisms lead to *DKK3* silencing remains unclear. Here, we analysed the promoter regions of *DKK3* by bioinformatics and found binding sites for MYCN. A dual‐luciferase reporter gene assay and ChIP experiments revealed that MYCN negatively regulates *DKK3* at the transcriptional level in B‐ALL cell lines, and using bisulphite sequencing PCR, we affirmed that *MYCN* has no effect on the methylation of the *DKK3* promoter. *MYCN* silencing in B‐ALL cells resulted in reduced cell proliferation, increased apoptosis and G1 phase arrest. Treatment with *MYCN* siRNA or 5‐aza‐2′‐deoxycytidine (5‐AdC), a demethylating agent, significantly increased the levels of DKK3 mRNA and protein and decreased the protein levels of p‐GSK3β and nuclear β‐catenin, which indicates inhibition of the Wnt/β‐catenin pathway in vitro. *MYCN* knockdown significantly decreased the tumorigenic capacity of Nalm6 cells, which restored DKK3 levels and inhibited the Wnt/β‐catenin pathway in vivo. Our study provides an increased understanding of adult B‐ALL pathogenesis, which may be beneficial to the development of effective prognostic markers or therapeutic targets.

## 1 INTRODUCTION

Despite ongoing improvements in the outcomes of patients with acute lymphoblastic leukaemia (ALL), only 30%‐40% of adult B‐cell ALL (B‐ALL) patients achieve long‐term remission due to its aggressive biological behaviour, even with allogeneic haematopoietic stem cell transplantation and chimeric antigen receptor T‐cell therapy.^1^, ^2^, ^3^, ^4^, ^5^, ^6^ Therefore, it is very important to understand the underlying mechanisms of adult B‐cell ALL carcinogenesis and progression to develop novel therapeutic targets and optimal treatment strategies for adult B‐ALL patients.

*DKK3* is a member of the *Dickkopf* (*DKK*) gene family and is a putative Wnt antagonist. *DKK3* may function as an anti‐oncogene, as it induces apoptosis and regulates the Wnt signalling pathway during tumorigenesis.^7^, ^8^, ^9^ *DKK3* gene expression is frequently down‐regulated by promoter hypermethylation in many solid tumours and haematological malignancies.^8^, ^10^, ^11^ Moreover, some miRNAs can also down‐regulate the expression of *DKK3* at the post‐transcriptional level in some cancers.^9^, ^12^, ^13^ Indeed, in support of this, our previous studies have shown that *miR‐708* down‐regulates the expression and secretion of DKK3‐induced activation of Wnt/β‐catenin pathways in B‐ALL cell lines through direct targeting of the 3′‐UTR of *DKK3*.^14^ To determine whether transcription factors lead to *DKK3* silencing, we analysed the promoter regions of *DKK3* by bioinformatics and found multiple binding sites for MYCN, which indicates that the expression of *DKK3* may be down‐regulated by MYCN at the transcriptional level.

*MYCN*, a member of the *MYC* proto‐oncogene family, encodes a nuclear transcriptional activator/repressor phosphoprotein that functions in the direct up‐ or down‐regulation of genes via promoter binding. *MYCN* also acts through indirect pathways to control cell proliferation, apoptosis and differentiation; *MYCN* is also extensively involved in oncogenesis.^15^, ^16^, ^17^, ^18^ *MYCN* is overexpressed in many malignancies, such as retinoblastoma, medulloblastoma and neuroblastoma, and *MYCN* overexpression is correlated with increased growth potential and poor prognosis.^19^, ^20^, ^21^ However, few studies to date have shown that *MYCN* can promote cell proliferation and inhibit the activity of tumour suppressor gene‐related signalling pathways that participate in adult B‐ALL, which cumulatively lead to a poor prognosis.

Here, we show that the *MYCN* mRNA level is negatively correlated with *DKK3* mRNA in adult B‐ALL patient samples. Moreover, our data revealed that MYCN binds directly to the promoter region of *DKK3* in B‐ALL cell lines. We also further determined that MYCN can directly down‐regulate DKK3 expression at the transcriptional level to activate Wnt/β‐catenin signalling, which in turn leads to proliferation of B‐ALL cell lines. Moreover, *MYCN* knockdown was shown to significantly inhibit cell proliferation and tumour growth in vitro and in vivo. Therefore, our results demonstrate that the targeting of *MYCN* upstream restores the high expression of DKK3 and may be a new treatment strategy for adult B‐ALL.

## 2 MATERIALS AND METHODS

### 2.1 Patient samples

We studied 12 matched samples of adult B‐ALL obtained at initial diagnosis, complete remission (CR) and after relapse from patients in the Department of Hematology of the First Affiliated Hospital of Harbin Medical University. The diagnosis was established according to the WHO diagnostic criteria.^22^ This study was approved by the Ethics Committee of Human Experimentation at Harbin Medical University. Informed consent was provided in accordance with the Declaration of Helsinki. Detailed patient information is described in Table S1. Bone marrow mononuclear cells from the patients and normal CD19^+^ B cells from the bone marrow of healthy volunteers (normal B cells) were obtained as previously reported.^14^

### 2.2 Reagents

5‐Aza‐2′‐deoxycytidine (5‐AdC) was purchased from Sigma (Sigma‐Aldrich Corporation, St. Louis, MO, USA), dissolved in 100% DMSO to generate a stock concentration of 10^−2^ M, stored at −20°C and diluted to the desired concentration in RPMI 1640 before use.

### 2.3 Cell culture

The human B‐ALL cell lines Nalm6 and BALL‐1 were used in this study. The characteristics and the culture conditions are described in the Supporting information.

### 2.4 Bisulphite sequencing PCR

We performed BSP as previously described.^14^ Five to ten clones from each sample were subjected to cycle sequencing (PE Applied Biosystems, Warrington, UK) and analysed using an ABI 310 sequencer (Applied Biosystems, Foster City, CA, USA). The primers used for BSP and the details of these experiments are given in the Supporting information.

### 2.5 Dual‐luciferase gene reporter assay

Luciferase assays were performed in Nalm6 cells. Luciferase activity was measured in the transfected cells using a Dual‐Luciferase Reporter Assay System (Promega, WI, USA).

### 2.6 Chromatin immunoprecipitation (ChIP) assay

ChIP analysis was performed according to the manufacturer's instructions (ChIP kit; Upstate Biotechnology, Waltham, USA) using an anti‐MYCN antibody (Becton Dickinson Pharmingen, San Diego, USA). *DKK3* promoter‐specific primers and detailed methods are included in the Supporting information.

### 2.7 Cell proliferation analysis

Cell proliferation was assayed using the Cell Counting Kit‐8 method (CCK‐8; Sigma‐Aldrich).

### 2.8 Flow cytometric analysis of the cell cycle and apoptosis

For the cell cycle analysis, the cells were stained with propidium iodide (PI, Sigma‐Aldrich). The apoptosis analysis was performed using Annexin V‐FITC/PI according to the manufacturer's protocol (Sigma‐Aldrich).

### 2.9 In vivo experiments

In vivo experiments performed in NOD/SCID mice are described in the Supporting information.

### 2.10 Statistical analyses

The data are presented as the means ± SD. Comparisons of two or more data sets were analysed using one‐way analysis of variance (ANOVA) followed by Tukey's multiple comparisons test. The size of the tumours from the in vivo experiments and the cell proliferation analysis from the in vitro experiments were analysed by two‐way ANOVA followed by Bonferroni's multiple comparisons test. The correlation analysis between *MYCN* and *DKK3* mRNA expression was performed using Spearman's correlation analysis. Values were considered significant at *P* < .05. All analyses were performed using GraphPad Prism 5.0 (GraphPad Software, USA). Additional methods and details are described in the Supporting information.

## 3 RESULTS

### 3.1 MYCN and DKK3 mRNA expression in adult B‐ALL patients and cell lines

**Figure 1:**
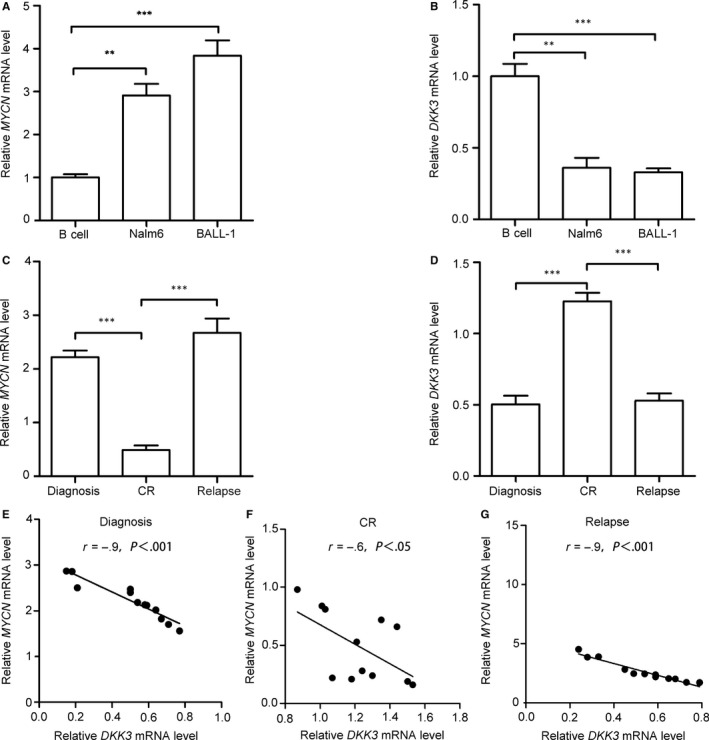
*MYCN* and *DKK3* mRNA expression in B‐cell acute lymphoblastic leukaemia (B‐ALL) cell lines and adult B‐ALL patient samples. Compared with normal B cells, *MYCN* mRNA (A) was highly expressed, and *DKK3* mRNA (B) was expressed at low levels in B‐ALL cell lines. (C) *MYCN* mRNA expression was high in adult B‐ALL samples obtained at initial diagnosis and at relapse (n = 12) compared with patient samples obtained after complete remission (CR). (D) *DKK3* mRNA expression was low in adult B‐ALL samples obtained at initial diagnosis and at relapse (n = 12) compared with patient samples obtained after CR. The *MYCN* and *DKK3* mRNA expression levels were negatively correlated in adult B‐ALL patient samples obtained at initial diagnosis (E), CR (F) and relapse (G). *MYCN* and *DKK3* mRNA levels were measured by quantitative real‐time PCR (qRT‐PCR), and the data are presented as the means ± SD from three separate experiments. **P < .01; ***P < .001

To analyse the relationship between *MYCN* and *DKK3* expression, we performed quantitative real‐time PCR (qRT‐PCR) and assayed *MYCN* and *DKK3* mRNA levels in adult B‐ALL patients and cell lines. We detected higher *MYCN* mRNA levels in Nalm6 and BALL‐1 cells than in normal B cells (Figure 1A). *MYCN* mRNA expression was remarkably increased in paired samples obtained at initial diagnosis and relapse compared with matched adult B‐ALL patient samples obtained after CR (Figure 1C). Subsequently, we examined *DKK3* mRNA expression in paired adult B‐ALL patient samples and cell lines. Compared with normal B cells, *DKK3* mRNA expression was lower in Nalm6 and BALL‐1 cells (Figure 1B). The levels of *DKK3* mRNA in samples obtained from adult B‐ALL patients at initial diagnosis and after relapse were lower than those in samples from the same patients after a CR was achieved (Figure 1D). Next, Spearman's correlation analysis showed that *MYCN* mRNA expression was negatively correlated with *DKK3* mRNA in adult B‐ALL patients (Figure 1E,F,G).

### 3.2 MYCN directly binds to the DKK3 promoter but has no effect on DKK3 promoter methylation

**Figure 2:**
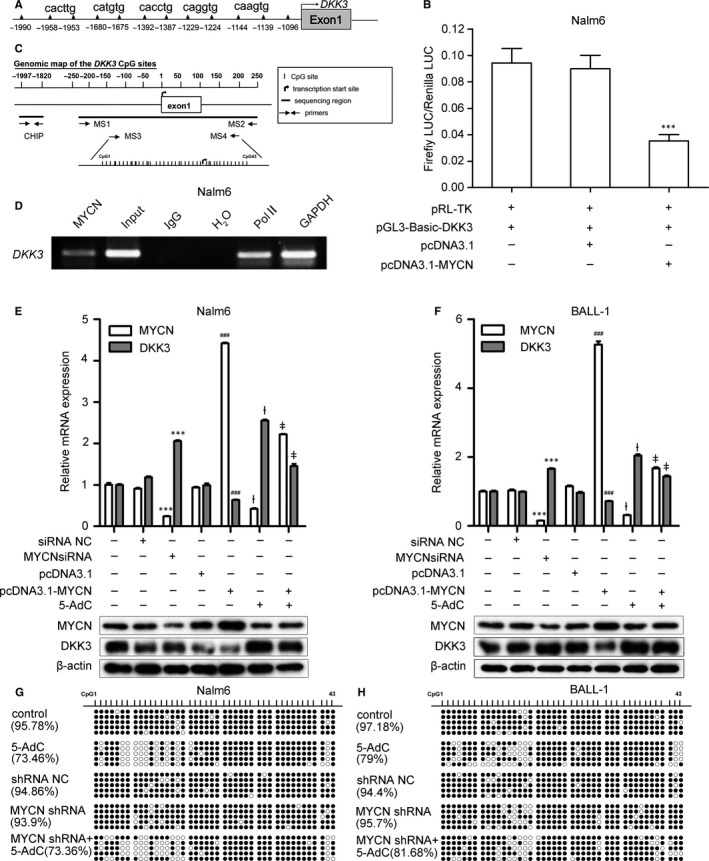
MYCN directly binds to the *DKK3* promoter but has no effect on *DKK3* promoter methylation. (A) Schematic of the putative *DKK3* promoter region containing five potential binding sites for MYCN. (B) Luciferase assays show that the relative luciferase activity decreased in Nalm6 cells co‐transfected with pcDNA3.1‐MYCN and pGL3‐Basic‐DKK3. ***P < .001 vs. pRL‐TK+pGL‐Basic‐DKK3 + pcDNA3.1. (C) CpG site distribution in the promoter region of *DKK3* and locations of the primers used in BSP and CHIP assays.(D) Chromatin immunoprecipitation (ChIP) analysis using an anti‐MYCN antibody shows that MYCN was bound to the *DKK3* promoter region. (E, F) MYCN and DKK3 mRNA (top) and protein (bottom) expression levels changed after transfection with pcDNA3.1‐*MYCN* or *MYCN* siRNA and/or treatment with 5‐AdC in Nalm6 and BALL‐1 cells. The *MYCN* and *DKK3* mRNA levels were measured by quantitative real‐time PCR (qRT‐PCR). MYCN and DKK3 protein expression was measured by Western blot, and β‐actin was used as a loading control. (G, H) After treatment with *MYCN* shRNA and/or 5‐AdC, the methylation status of the promoter region of *DKK3* in CpG islands in B‐ALL cell lines was determined by bisulphite sequencing PCR (BSP). The percentage of methylation was determined via the ratios of methylated cytosine in 5 to 10 sequenced clones. The solid spots indicate methylated CpG dinucleotides; the hollow spots indicate unmethylated CpG dinucleotides. All regions are shown relative to the transcription start site (TSS). Each experiment was repeated three times. The data are presented as the means ± SD. ***P < .001 vs. siRNA NC; ^###^ *P* < .001 vs. pcDNA3.1; ^ƚ^ *P* < .001 vs. control; ^ǂ^ *P* < .001 vs. 5‐AdC. Non‐transfected cells were used as a control. NC: negative control

To elucidate the relationship between *MYCN* and *DKK3* in adult B‐ALL, we constructed firefly luciferase reporters containing the *DKK3* gene promoter region and predicted MYCN binding sites (Figure 2A). Co‐transfection of Nalm6 cells with a *MYCN* overexpression plasmid greatly reduced the luciferase activity driven by the *DKK3* promoter region (Figure 2B). Next, a ChIP analysis was performed to further investigate whether MYCN binds to the promoter region of *DKK3* in Nalm6 cells. As shown in Figure 2D, DNA sequence fragments from the *DKK3* promoter onto which MYCN was recruited were amplified by PCR using specific primers. In addition, *MYCN* overexpression significantly decreased the DKK3 mRNA and protein levels and *MYCN* siRNA increased DKK3 mRNA and protein expression in Nalm6 and BALL‐1 cells (Figure 2E,F). These data indicated that MYCN binds directly to the promoter region of *DKK3* and significantly down‐regulates DKK3 mRNA and protein expression.

*DKK3* is reported to be silenced by promoter CpG methylation in ALL.^11^, ^23^ In this study, 5‐AdC treatment increased DKK3 mRNA and protein expression in both cell lines. However, MYCN mRNA and protein levels were significantly decreased in Nalm6 and BALL‐1 cells after 5‐AdC treatment. Interestingly, when *MYCN*‐overexpressing Nalm6 and BALL‐1 cells were treated with 5‐AdC, DKK3 mRNA and protein levels were decreased compared with 5‐AdC treatment alone (Figure 2E,F). To further determine whether MYCN expression was associated with *DKK3* promoter methylation, we silenced *MYCN* in Nalm6 and BALL‐1 cells and examined DNA methylation at 43 CpG sites in the *DKK3* promoter region via BSP (Figure 2C). No significant difference was observed between the *MYCN* shRNA and the negative control shRNA groups (Figure 2G,H). These results suggest no significant involvement of MYCN in the maintenance of *DKK3* methylation and that *MYCN* knockdown and 5‐AdC increase DKK3 expression through two independent mechanisms.

### 3.3 MYCN depletion reduces proliferation and increases apoptosis of B‐ALL cells and restores the DKK3‐mediated inhibition of the Wnt/β‐catenin pathway

**Figure 3:**
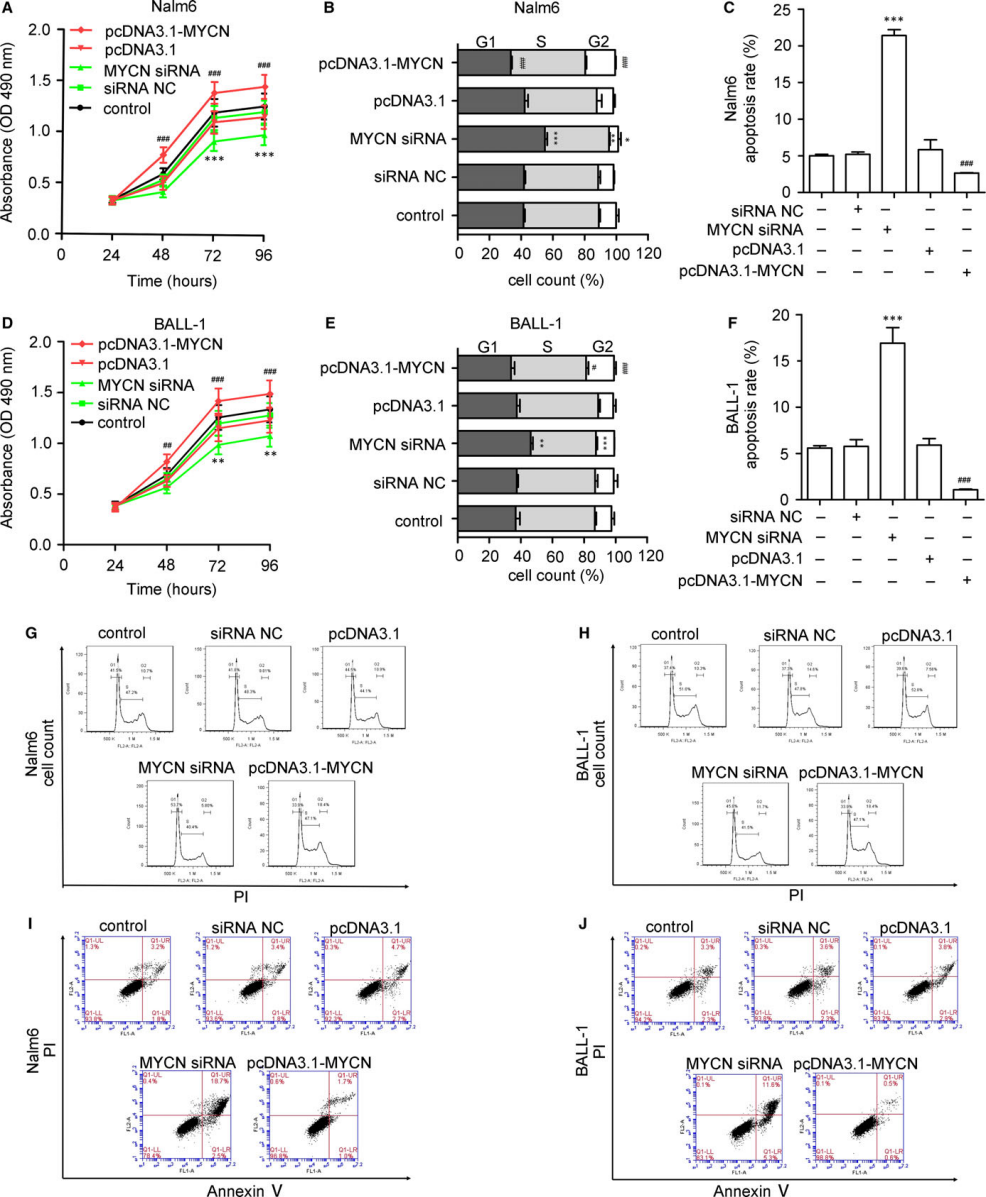
Influence of MYCN expression on cell proliferation, the cell cycle and apoptosis. (A, D) *MYCN* siRNA significantly decreased the proliferation rates of Nalm6 and BALL‐1 cells as measured by CCK‐8 assays. *MYCN* siRNA treatment significantly increased the proportions of Nalm6 and BALL‐1 cells in G1 phase, as measured by flow cytometry after PI staining. Histograms (B, E) and representative flow cytometry plots (G, H) of cell cycle alterations. *MYCN* siRNA treatment increased the number of apoptotic Nalm6 and BALL‐1 cells, as measured by flow cytometry after annexin V/PI staining. Histograms (C, F) and representative flow cytometry plots (I, J) of cell apoptosis alterations. LR: early apoptotic cells; UR: late apoptotic cells. The numbers represent the combined percentages of apoptotic cells in the LR and UR quadrants. Non‐transfected cells were used as a control. The data are presented as the means ± SD from three separate experiments. *P < 0.05 vs. siRNA; **P < 0.01 vs. siRNA; ***P < .001 vs. siRNA NC; ^#^ *P* < 0.05 vs. pcDNA3.1; ^##^ *P* < 0.1 vs. pcDNA3.1; ^###^ *P* < .001 vs. pcDNA3.1. NC: negative control

**Figure 4:**
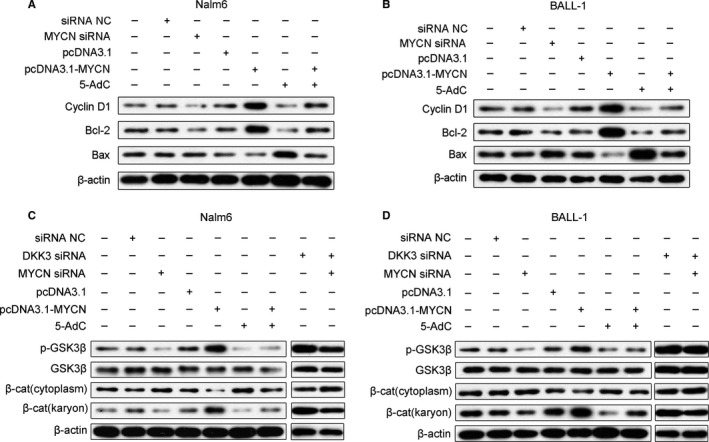
Effects of MYCN and 5‐AdC on the expression of proteins related to the cell cycle, apoptosis and the Wnt/β‐catenin signalling pathway. Nalm6 and BALL‐1 cells were transfected with *MYCN* siRNA or pcDNA3.1‐*MYCN* and/or treated with 5‐AdC. (A, B) Representative Western blots show the expression of cyclin D1, Bcl‐2 and Bax in Nalm6 and BALL‐1 cells. (C, D) Representative Western blots show the expression of p‐GSK3β, GSK3β and β‐catenin (cytoplasmic and nuclear) in Nalm6 and BALL‐1 cells. Untreated cells were used as a control, and the protein levels were measured by Western blots from three separate experiments; β‐actin was used as a loading control. NC: negative control, β‐cat: β‐catenin

To examine the effects of *MYCN* expression on leukemogenesis, we evaluated the effects of *MYCN* on cell proliferation and the induction of apoptosis in B‐ALL cell lines. As shown in Figure 3A,D, *MYCN* siRNA inhibited cell proliferation, whereas *MYCN* overexpression promoted proliferation of Nalm6 and BALL‐1 cells. To evaluate the mechanism of *MYCN* inhibition in the suppression of cell proliferation, the cell cycle distribution and cell apoptosis were examined by flow cytometry. After cells were treated with *MYCN* siRNA, the proportions of Nalm6 cells (Figure 3B,G) and BALL‐1 cells (Figure 3E,H) in G1 phase increased from 41.8% to 53.7% and from 37.3% to 45.8%, respectively. In addition, apoptosis assays revealed that *MYCN* siRNA treatment increased apoptosis but that *MYCN* overexpression decreased apoptosis of Nalm6 (Figure 3C,I) and BALL‐1 (Figure 3F,J) cells. Next, we examined the cell cycle and apoptosis‐relevant proteins cyclin D1, Bcl‐2 and Bax via Western blotting (Figure 4A,B). *MYCN* siRNA decreased cyclin D1 and Bcl‐2 expression and increased Bax expression in these cell lines. These results demonstrated that the inhibition of cell proliferation by *MYCN* depletion is most likely mediated by G1 cell cycle arrest and apoptosis.

MYCN can directly down‐regulate DKK3 expression (Figure 2). DKK3 is reported to be a putative antagonist of the Wnt/β‐catenin signalling pathway,^7^ and activation of this pathway has been implicated in the pathogenesis of leukaemia.^24^, ^25^, ^26^ To explore the effects of MYCN on the Wnt/β‐catenin signalling pathway, we measured the protein levels of p‐GSK3β, GSK3β and both cytoplasmic and nuclear β‐catenin in Nalm6 and BALL‐1 cells by Western blotting. In contrast to DKK3 protein levels (Figure 2E,F), the p‐GSK3β and nuclear β‐catenin protein levels were increased in *MYCN*‐overexpressing cells. However, p‐GSK3β and nuclear β‐catenin expression were decreased after *MYCN* siRNA and 5‐AdC treatment (Figure 4C,D). These results suggested that *MYCN* activates the Wnt/β‐catenin signalling pathway but that *MYCN* siRNA or 5‐AdC suppresses this pathway. Interestingly, knockdown of both *MYCN* and *DKK3* increased p‐GSK3β and nuclear β‐catenin protein levels, suggesting activation of the Wnt/β‐catenin pathway. When *MYCN*‐overexpressing Nalm6 and BALL‐1 cells were treated with 5‐AdC, nuclear β‐catenin and p‐GSK3β protein levels were increased compared with 5‐AdC treatment alone (Figure 4C,D).

### 3.4 MYCN shRNA exerts antitumour effects in mice with Nalm6 cell xenografts

**Figure 5:**
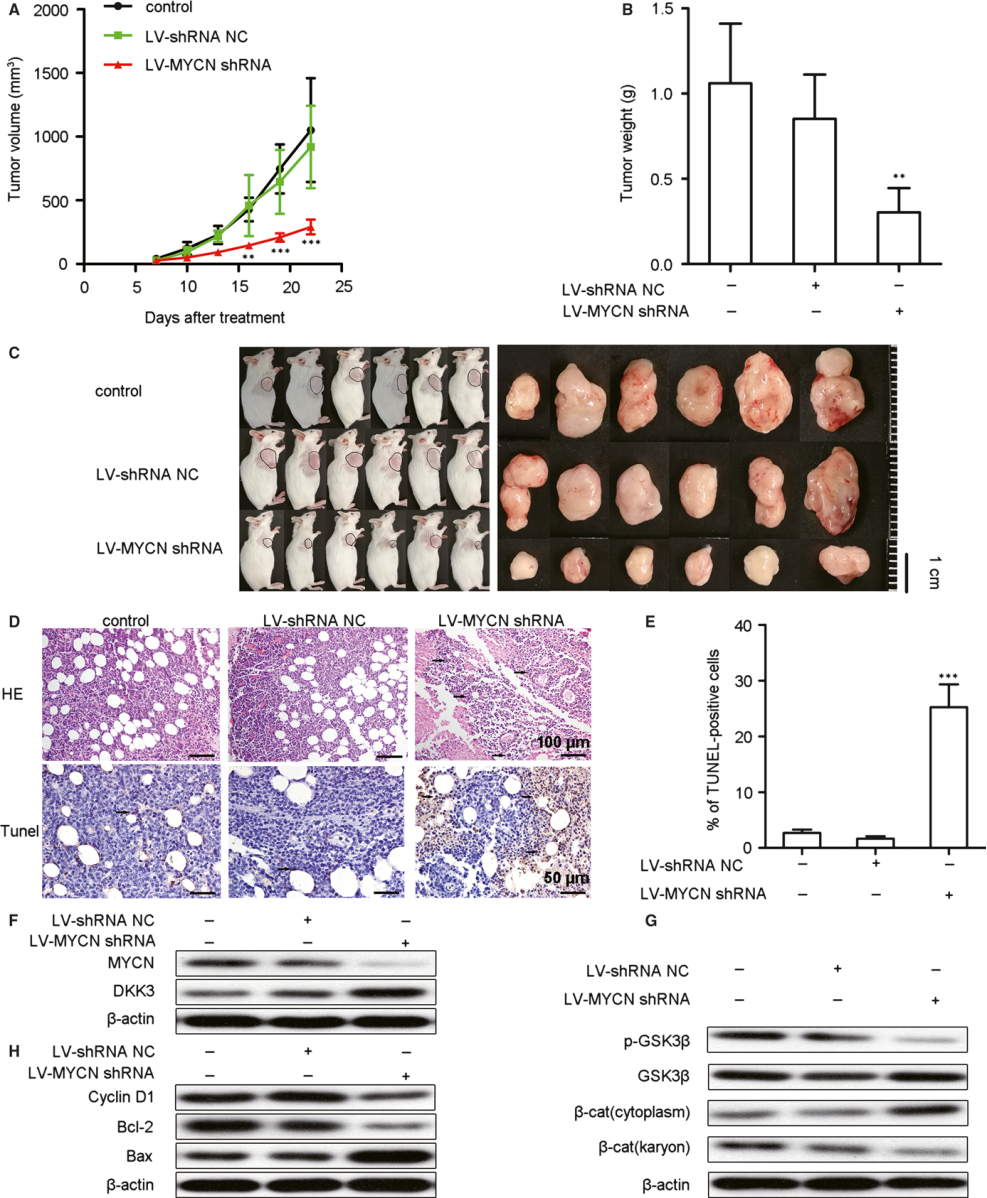
*MYCN* shRNA suppresses tumour growth via the restoration of DKK3‐mediated inhibition of the Wnt/β‐catenin pathway in a murine Nalm6 xenograft model. Mice burdened with growing Nalm6 tumours were infected with Lv‐shRNA NC or Lv‐*MYCN* shRNA or left untreated (control), and the tumour sizes (A) were measured for 22 days (n = 6). The tumour weights (B) were also measured after 22 days (n = 6). Images of tumour‐bearing mice (C) show that the tumours were smaller in *MYCN* shRNA‐treated animals. (D) Haematoxylin and eosin (H&E) (upper) and TUNEL (lower) staining of the xenograft tumour tissues from 3 mice of each group. In the H&E‐stained sections, the original magnification was 200 × . In the TUNEL‐stained sections, positive cells are indicated by brown staining, with an original magnification of 400 × . Representative fields are shown. Arrows indicate tumour cells. (E) The TUNEL‐positive cells were measured from 3 randomly chosen fields (3 × 10^4^ μm^2^/field; n = 9). (F) Representative Western blots show the protein expression of MYCN and DKK3. (G) Representative Western blots show the expression of p‐GSK3β, GSK3β and β‐catenin (cytoplasmic and nuclear). (H) Representative Western blots show the expression of Bax, Bcl‐2 and cyclin D1. Expression of proteins was measured by Western blotting, and β‐actin was used as a loading control; we used 3 mice from each group. All data are presented as the means ± SD. **P < .01 vs. Lv‐shRNA NC; ***P < .001 vs. Lv‐shRNA NC. NC: negative control, β‐cat: β‐catenin

To assess the potential of *MYCN* shRNA therapy in vivo, we tested the antitumour effects of *MYCN* shRNA in a mouse model. During the 22‐day observation period, tumour growth was inhibited in mice treated with *MYCN* shRNA (Figure 5A). At the end of the 22 days, the tumour weights (Figure 5B) and the tumour volumes (Figure 5C) were significantly lower in the *MYCN* shRNA‐treated mice. The morphologies of Nalm6 tumour xenograft cells were examined after H&E staining. A greater percentage of the tumour cells derived from *MYCN* shRNA‐treated mice exhibited characteristics of apoptosis, such as cell volume shrinkage, nuclear pyknosis and prominent apoptotic bodies (Figure 5D). The number of TUNEL‐positive cells significantly increased (Figure 5D,E) upon *MYCN* shRNA treatment, which suggested that *MYCN* shRNA induces apoptosis in vivo.

To further address the mechanisms that underlie the effects of *MYCN* shRNA, we analysed proteins downstream of Wnt/β‐catenin signalling and apoptosis‐relevant proteins by Western blotting. In contrast to the increased DKK3 protein levels in murine tumour tissues (Figure 5F), both p‐GSK3β and nuclear β‐catenin expression decreased after *MYCN* shRNA treatment (Figure 5G). The suppression of *MYCN* expression increased the levels of Bax and decreased the levels of Bcl‐2 and cyclin D1 proteins in cell‐derived tumour xenografts (Figure 5H). These findings suggested that *MYCN* shRNA inhibits the Wnt/β‐catenin signalling pathway, enhances apoptosis and restores DKK3 levels in vivo.

## 4 DISCUSSION

In this study, we identified *MYCN* as a strong marker for the onset and progression of adult B‐ALL. We provide evidence that *MYCN* expression is markedly up‐regulated in newly diagnosed and relapsed adult B‐ALL but is down‐regulated in patients who achieve CR. MYCN overexpression has been reported in haematologic malignancies, such as lymphoma,^27^ chronic lymphocytic leukaemia (CLL) 28 and paediatric T‐ALL,^29^ and it is considered a well‐established marker of a poor prognosis in these diseases. In addition, a previous study showed that MYCN overexpression rapidly led to acute myeloid leukaemia (AML) in mice.^30^ MYCN was able to induce pre‐B‐ALL/LBL directly from the progenitor B cells of mice in the absence of *Ink4a* and *Arf*.^31^ In our experiments, *MYCN* promoted cell proliferation and inhibited apoptosis. However, *MYCN* knockdown significantly inhibited tumour growth and promoted apoptosis in vivo and in vitro. This finding suggests that *MYCN* plays a proto‐oncogenic role and indicates that MYCN is a potential component of adult B‐ALL pathogenesis and may therefore be a viable candidate for targeted therapy.

The reduced expression of DKK3 has become a hallmark of several haematologic malignancies, such as ALL, CLL, AML and myelodysplastic syndrome, and its down‐regulation is associated with a poor prognosis in patients.^11^, ^32^, ^33^, ^34^ *DKK3* promoter hypermethylation is associated with *DKK3* silencing in ALL cells, and DKK3 expression was restored after exposure to 5‐AdC, which indicates that hypermethylation is one of the mechanisms by which *DKK3* is silenced in ALL cells.^11^, ^23^ These previous results are consistent with our current work. However, when *MYCN*‐overexpressing Nalm6 and BALL‐1 cells were treated with 5‐AdC, DKK3 mRNA and protein levels decreased compared with 5‐AdC treatment alone, which indicates that methylation is not the only mechanism of *DKK3* silencing. MYCN might induce another mechanism of *DKK3* silencing such that 5‐AdC alone is unable to restore DKK3 expression. In previous studies, MYC has been shown to associate with DNA methyltransferases, which induce the transcriptional silencing of target genes in neuroblastoma^18^, ^35^, ^36^; this suggests that MYCN might play a similar role in the hypermethylation of *DKK3*. However, we found that the modulation of *MYCN* silencing had no effects on *DKK3* promoter methylation in B‐ALL cell lines. This finding indicates that MYCN might silence *DKK3* gene expression but not via methylation.

In this study, we also found a low *DKK3* expression level in B‐ALL cell lines and in patient samples from adult B‐ALL obtained at initial diagnosis and at relapse. Our results show that *DKK3* may be a tumour suppressor in adult B‐ALL. DKK3 may contribute to the suppression of tumours by virtue of its ability to antagonize Wnt signalling. The Wnt/β‐catenin pathway plays a crucial role in haematopoietic differentiation,^37^ and aberrant activation Wnt/β‐catenin signalling has been linked to haematologic malignancies, including AML and ALL.^26^, ^38^, ^39^ Therefore, it can be speculated that the silencing of *DKK3* gene expression leads to the activation of the Wnt/β‐catenin signalling pathway, which is involved in cancer development. In this study, the knockdown of *MYCN* suppressed the Wnt/β‐catenin signalling pathway in vitro and in vivo. Depletion of DKK3 by siRNA eliminated the inhibition effect of MYCN siRNA on the Wnt/β‐catenin pathway in vitro. These results demonstrated that *MYCN* siRNA restored the DKK3‐mediated inhibition of the Wnt/β‐catenin signalling pathway. When *MYCN*‐overexpressing Nalm6 and BALL‐1 cells were treated with 5‐AdC, the nuclear β‐catenin and p‐GSK3β protein levels remained increased, which suggests that 5‐AdC cannot by itself suppress the Wnt/β‐catenin signalling pathway in vitro. The suppression of MYCN expression may be another key factor for the inhibition of the Wnt/β‐catenin signalling pathway, and this effect may result from the restoration of DKK3 levels.

In this manuscript, we also found that MYCN directly binds to the *DKK3* promoter; furthermore, MYCN overexpression significantly down‐regulated DKK3 mRNA and protein levels in two B‐ALL cell lines. This finding suggests that *MYCN* may regulate *DKK3* at both the transcriptional and translational levels. DKK3 expression has previously been related to *MYCN* and *c‐MYC* gene expression levels.^40^, ^41^ We confirmed the previously reported inverse relationship between *DKK3* and *MYCN* gene expression in neuroblastic cell lines.^40^ *MYCN* has been shown to directly bind to the promoter of some genes to drive transcription; these genes include *SKP2*,* NDRG1*,* TG2* and *HMGA1*.^17^, ^42^ However, previous studies did not find a direct relationship between *MYCN* and *DKK3*. Here, we confirmed direct targeted down‐regulation of *DKK3* by MYCN in B‐ALL cell lines, which is a strong indication that this newly discovered molecular interaction between MYCN and the upstream Wnt/β‐catenin pathway is relevant to the biology of adult B‐ALL.

Our analyses therefore show that MYCN directly down‐regulates *DKK3*, which results in Wnt/β‐catenin signalling pathway activation. This novel regulatory cascade might function in the regulation of the Wnt/β‐catenin signalling pathway, which is involved in the genesis and development of adult B‐ALL. The inhibition of this pathway by DKK3 and the release of this MYCN‐mediated suppression might therefore have important biological consequences in the prevention and treatment of adult B‐ALL. However, due to the small number of patients in our study, we could not definitively address whether MYCN has any predictive value for the prognosis of adult B‐ALL. Therefore, we analysed published gene expression data from The Cancer Genome Atlas and Gene Expression Omnibus, but we did not find abnormal *MYCN* expression or prognosis‐related data for adult B‐ALL. Thus, further research with a larger cohort will be needed to assess whether MYCN is predictive for adult B‐ALL relapse. In addition, the HDAC inhibitor vorinostat has been shown to down‐regulate MYCN mRNA and protein levels in neuroblastoma.^43^, ^44^, ^45^ In the present study, we confirmed that 5‐AdC has a similar effect. We therefore believe that siRNA either alone or in combination with epigenetic drugs may be of potential therapeutic significance for *MYCN*‐amplified adult B‐ALL.

## CONFLICT OF INTEREST

The authors have no conflict of interest to declare.
