# Supplementary material for: MYCN is a novel oncogenic target in adult B‐ALL that activates the Wnt/β‐catenin pathway by suppressing DKK3
Source: J Cell Mol Med. 2018 Apr 19;22(7):3627–37. doi: 10.1111/jcmm.13644 (PMC6010754; doi:10.1111/jcmm.13644)
Supplement: Supplementary file 2 [file JCMM-22-3627-s002.doc]

**SUPPLEMENTAL INFORMATION**

***MYCN* is a novel oncogenic target in adult B-ALL that activates the** **Wnt/β-catenin pathway by suppressing *DKK3***

**Supplemental Methods and Materials**

**Cell culture**

The human B-ALL cell lines Nalm6 and BALL-1 were kindly provided by the Shanghai Cell Bank, Chinese Academy of Sciences (Shanghai, China). The cells were maintained in RPMI 1640 culture medium supplemented with 10% fetal bovine serum (FBS), 100 U/mL penicillin and 100 U/mL streptomycin, and incubated in a 5% CO_2_-humidified incubator at 37°C. The untransfected cells and the cells transfected after 24 h with a *MYCN*-overexpression plasmid were treated with 10 μmol/L 5-AdC for 48 h and then harvested. Alternatively, the cells were transfected with a *MYCN*-overexpression plasmid or *MYCN* siRNA or co-transfected with a *MYCN* siRNA and *DKK3* siRNA and then harvested.

**siRNA and plasmid transfection**

Cells were plated in growth medium without antibiotics approximately 24 h before transfection. The transient transfection of plasmids/siRNA (Invitrogen, USA) was performed using Attractene transfection reagent (QIAGEN, Germany) according to the manufacturer’s protocol. The sequence of *MYCN* siRNA was 5’-CAGCAGCAGUUGCUAAAGATT-3’, the sequence of *DKK3* siRNA was 5’-AACAACCAGACUGGACAAATT-3’, and the sequence of the negative control siRNA was 5’-UUCUCCGAACGUGUCACGUTT-3’. The cells were harvested at the indicated time points.

**shRNA constructs and lentiviral infection**

*MYCN* short hairpin RNA (shRNA) constructs cloned in the pLKO.1 puro vector were designed by the ShangHai GenePharma Co., Ltd. The sequence of *MYCN* shRNA was 5’-CAGCAGCAGUUGCUAAAGATT-3’, and the sequence of negative control shRNA was 5’-CAGCAGCAGUUGCUAAAGATT-3’. Lentiviral production and infection were performed as specified by Sigma-Genosys using MISSION Lentiviral Packaging Mix. The transduced Nalm6 cells were selected in puromycin (2 μg/ml) for two weeks. After puromycin selection, *in vivo* experiments were performed.

**Bisulfite sequencing**

We performed the bisulfite modification analysis of genomic DNA as described previously[^26^](#_ENREF_26). The bisulfite sequencing PCR (BSP) reaction system was composed of 1× PCR buffer (0.25 mM KCl), 6.25 μM dNTP mix, 0.5 μM of each of the primers, 0.75 U of the hot start DNA polymerase (TaKaRa, Tokyo, Japan), and 20 ng of the modified DNA (bisulfite-converted as described above). The first-round PCR consisted of 35 cycles at an annealing temperature of 56°C with the use of the primers for MS1, 5’-AGGTGAGGAGTAGAGTTTAGTTTG-3’, and MS2, 5’-TCCTCCATCAATTCCTCAACC -3’. With the use of one twentieth of the first-round PCR product as a template, the second-round PCR consisted of 35 cycles at an annealing temperature of 60°C with the primers MS3, 5’-TTCGGGTGTAGGGGAGTTG-3’, and MS4, 5’-TCTCATTAAAAATAACCTCCTCC-3’. Under these conditions, we could assess the DNA methylation status of the *DKK3* gene, which has a length of 342 bp and contains 43 CpG sites relevant to the TSS. The PCR products were analyzed on a 1.5% agarose gel. Each purified product was cloned into the pMD19-T Vector (TaKaRa) and transfected into DH5α competent cells (Vazyme Biotech Co., Piscataway, NJ, USA). Five to ten clones from each sample were subjected to cycle sequencing (PE Applied Biosystems, Warrington, UK) and analyzed using an ABI 310 sequencer (Applied Biosystems, Foster City, CA, USA).

**Luciferase reporter plasmid constructs**

The [promoter](../../../../C:%5CUsers%5Clenovo%5CAppData%5CLocal%5Cyoudao%5Cdict%5CApplication%5C7.5.2.0%5Cresultui%5Cdict%5C%3Fkeyword=promoter)[sequence](../../../../C:%5CUsers%5Clenovo%5CAppData%5CLocal%5Cyoudao%5Cdict%5CApplication%5C7.5.2.0%5Cresultui%5Cdict%5C%3Fkeyword=sequence) of the *DKK3* gene was available in The UCSC Genome Browser database ([http://genome.ucsc.edu](http://genome.ucsc.edu/)), and the Consite website (<http://consite.genereg.net/cgi-bin/consite>) was used to predict the candidate promoter region and transcrip­tion factor binding sites (Figure 2A). A pair of primers was designed containing KpnI and Xho I sites to obtain the 895 bp promoter region (from -1990 to -1096 bp, with numbering relative to the transcription start site (TSS)) of the *DKK3* gene from human genomic DNA using PCR. The amplified products were purified and digested with restriction endonuclease and then cloned into a pGL3-basicvector (Promega, USA). The resulting construct was named pGL3-Basic-*DKK3* and was verified by sequencing. The sequences of the *DKK3* promoter primers were (forward) 5’-CGAGGTACCCCACACAAGAAAACAA-3’ and (reverse) 5’-GCTCGAGCTTAGTCTGCCGTGAT-3’. The underlined sequences indicate the restriction enzyme sites for KpnI and Xho I, respectively.

**Dual-luciferase gene reporter assay**

Nalm6 cells were seeded in 24-well plates at a density of 3×10^5^ cells/well and maintained in RPMI 1640 culture medium containing 10% FBS. For each well, 1 µg of pcDNA3.1*-MYCN* plasmid was co-transfected with 1 µg of luciferase reporter plasmid using the Attractene transfection reagent (4.5 μl/well, QIAGEN, Germany) according to the manufacturer's instructions; Renilla luciferase construct (10 ng) (pRL-TK, Promega, USA) was co-transfected as an internal reference control, and pGL3-Basic (Promega, USA) was used as a negative control. At 48 h after transfection, luciferase activity was measured using a Dual-Luciferase Reporter Assay System (Promega, USA), and the relative luciferase intensity was determined. Each experimental condition was measured in triplicate.

**Chromatin immunoprecipitation (ChIP) assay**

ChIP analysis was performed according to the manufacturer’s instructions (ChIP kit; Upstate Biotechnology, Waltham, USA). Briefly, Nalm6 cells were incubated with 1% formaldehyde to cross-link the DNA-protein complexes. The crosslinks were heated at 37°C for 10 min. After being washed twice with cold PBS, approximately 1x10^6^ cells were lysed in 200 μl SDS lysis buffer (Upstate Biotechnology) containing protease inhibitors. Lysate was sonicated to shear the DNA to lengths between 200 and 1,000 bp. The range of DNA fragment size was confirmed by reversing the crosslinks with 8 μl 5 M NaCl in 200 μl cell lysate at 65°C for 4 h, recovering the DNA by phenol/chloroform extraction, and electrophoresing the sample on a 1% agarose/TBE gel. The sonicated cell lysate was diluted 10-fold in ChIP dilution buffer (Upstate), and 1% of the diluted DNA was kept as the input control for subsequent PCR analysis. Histones were pre-cleaned with 75 μl of salmon sperm DNA/protein A agarose 50% slurry for 30 min at 4°C with agitation. The cross-linked protein was then immunoprecipitated with 8 μg anti-MYCN antibody (Becton Dickinson Pharmingen, San Diego, USA), nonspecific IgG antibody (as the negative control of the antibody, Sigma, USA), or anti-RNA polymerase II antibody (positive control; Upstate Biotechnology) per sample overnight at 4°C with constant rotation. The antibody/histone complex was collected by adding 60 μl of salmon sperm DNA/protein A agarose slurry for 1 h at 4°C with constant rotation. The samples were washed and eluted according to the manufacturer’s instructions. Both the immunoprecipitated and the control input samples (each in 500 μl volume) were then subjected to cross-link reversal. To each sample was added 10 μl 0.5 M EDTA, 20 μl 1 M Tris-HCl, pH 6.5, and 2 μl of 10 mg/ml proteinase K, and samples were incubated for 1 h at 45°C. DNA was recovered by phenol/chloroform extraction and ethanol precipitation. PCR was performed with *DKK3* promoter-specific primers, *GAPDH* primers (positive control), and ddH_2_O (negative control). The forward and reverse primers were as follows: 5'- GTAGACTCCACACAAGAAAACAA-3' and 5'- CCTTCATGTTGCTGGCTTAA-3' (*DKK3*); and 5'-TACTAGCGGTTTTACGGGCG-3' and 5'- TCGAACAGGAGCAGAGAGCGA-3' (*GAPDH*).

**Cell proliferation analysis**

To evaluate cell proliferation, cells were seeded in a 96-well plate at a density of 1×10^4^ cells/well. A total of 10 μL of CCK-8 solution was added to 100 μL of culture medium. After the cells were incubated for 4 h at 37°C, the absorbance of the culture medium was measured at 450 nm (A450) using a scanning microplate spectrophotometer (Multiscan MK3, Thermo Fisher Scientific).

**Cell cycle and apoptosis analysis**

For cell cycle analysis, cells were fixed overnight in chilled methanol before staining with 50 μg/mL propidium iodide (PI, Sigma-Aldrich) in the presence of 1 mg/mL RNase (100 units/mL; Sigma-Aldrich) and 0.1% NP40 (Sigma-Aldrich).

For apoptosis analysis, samples were incubated with annexin V-fluorescein isothiocyanate (FITC)/PI according to the manufacturer’s protocol (Sigma-Aldrich). Cell-bound fluorescence was analyzed using a FACSCalibur flow cytometer (Becton Dickinson, CA, USA).

**RNA extraction and quantitative real-time PCR**

Total RNA was extracted using TRIzol reagent (Invitrogen); 1 µg was reverse-transcribed, and qRT-PCR was performed using an ABI PRISM 7900 sequence detection system (Biosystems). PCR amplification was performed in a reaction system containing cDNA, forward and reverse primers, 2X SYBR Green qPCR SuperMix, and distilled water. *DKK3* and *MYCN* transcript expression levels were measured by qRT-PCR using the forward primer 5’-TTTTCCACGCAGTTCTTTCC-3’ and the reverse primer 5’-TGAGCCTCTGAGATCCCTGA-3’ for *DKK3*, and the forward primer 5’-CTGAGCGATTCAGATGATGAAGATG-3’ and reverse primer 5’- GACAGCCTTGGTGTTGGAGGAG-3’ for *MYCN*. For transcript expression measurements, *β-actin* was used as an internal control using the forward primer 5'-CTTAGTTGCGTTACACCCTTTCTTG-3' and the reverse primer 5'-CTGTCACCTTCACCGTTCCAGTTT-3'. Experiments were repeated at least three times. The relative expression of the ampliﬁed RNA samples was calculated using the 2^-ΔΔCT^ method. The results are presented as the fold change of each mRNA relative to a control sample (CD19^+^ cells from a healthy volunteer).

**Western blot analysis**

Protein was extracted from cells, separated by SDS-PAGE and transferred onto polyvinylidene fluoride membranes. The membranes were blocked with 5% non-fat dry milk and incubated overnight at 4°C with primary antibodies against the following proteins: GSK3β, p-GSK3β (Ser 9), MYCN, DKK3, β-catenin, cyclin D1, Bcl-2, and Bax-1. These antibodies were purchased from Abcam (Shanghai, China). An anti-β-actin antibody obtained from Sigma-Aldrich was used as a loading control. The resulting bands on the immunoblots were visualized using a BCIP/NBT kit (Sigma, St. Louis, MO, USA). The band intensities from the Western blotting experiments were quantified with image analysis software (ImageQuant TL; Amersham Biosciences) prior to statistical analysis.

**Histological analysis**

Xenografted tumor tissues were fixed in 4% paraformaldehyde, embedded in paraffin, and then cut into 5-μm-thick sections. Sections were depleted of paraffin, rehydrated in a graded series of ethanol solutions, and then stained with hematoxylin and eosin (H&E) using a standard protocol and analyzed by light microscopy at 200x magnification.

**TUNEL assay**

DNA fragmentation was assessed by TUNEL staining using an In Situ Apoptosis Detection Kit according to the manufacturer’s instructions (Key GEN Bio TECH). Briefly, paraffin-embedded tumor tissues from mice were cut into 5-μm-thick sections and deparaffinized with xylene. The sections were incubated with 10 mg/mL Proteinase K for 15 min at room temperature and then washed with PBS for 5 min (3 times). Endogenous peroxidase was inactivated with 3% H_2_O_2_ for 10 min at room temperature, and the sections were then washed with PBS. Sections were immersed in terminal deoxynucleotidyl transferase (TdT) buffer, incubated at 37°C for 60 min (in a dark room), and then washed with PBS. The sections were incubated at room temperature for 30 min with anti-horseradish peroxidase-conjugated antibody, and the signals were visualized with diaminobenzidine. From each biopsy, at least three fields were evaluated. The apoptotic cells were stained brown and observed under light microscopy at 400x magnification (200×150 μm^2^/field).

***In vivo* experiments**

All animal procedures were performed under standard conditions in Harbin Medical University, and all experiments conformed to the animal care protocols of the institution. Nalm6 cells were infected with lentivirus encoding *MYCN* shRNA or empty vector negative control. A total of 18 male 4-6-week-old NOD/SCID mice, weighing 19.4±0.2 g and obtained from the Shanghai Institute of Drug of Chinese Academy of Sciences, were housed in specific pathogen-free conditions and randomly separated into three groups (n=6 per group). Each mouse was injected subcutaneously in the right flank region with uninfected or infected Nalm6 cells (1×10^7^ in 200 μL of PBS). Three groups were injected with the three different cell preparations: uninfected Nalm-6 cells; Lv-*MYCN* shRNA cells; and Lv-shRNA NC cells. One week later, the tumor sizes in the mice were measured every three days for 22 days. The tumor volume was calculated using an empirical formula: V[mm^3^]=1/2×[(the shortest diameter)^2^×(the longest diameter)]. All mice were sacrificed at the end of the 22-day observation period by cervical dislocation, and tumor specimens were collected, imaged, and measured for their volumes and weights. We also used tumor specimens from 3 mice of each group for TUNEL staining, H&E staining, and Western blot analysis. The expression of MYCN and DKK3 protein levels in the xenografted tumors was detected by Western blotting. Other markers for protein expression were also measured, including proteins downstream of Wnt/β-catenin signaling (e.g., cytoplasmic and nuclear β-catenin, p-GSK3β, and GSK3β), cell cycle proteins (e.g., cyclin D1), and cell apoptosis proteins (e.g., Bax, Bcl-2).
